# Supplementary material for: miRAW: A deep learning-based approach to predict microRNA targets by analyzing whole microRNA transcripts
Source: PLoS Comput Biol. 2018 Jul 13;14(7):e1006185. doi: 10.1371/journal.pcbi.1006185 (PMC6067737; doi:10.1371/journal.pcbi.1006185)
Supplement: S1 File — (PDF) [file pcbi.1006185.s001.pdf]

Supplementary Material

List of Figures

A Schematic of the procedure followed to generate training and testing data. 2
B The number of flanking upstream/downstream nucleotides conditions the performance of the network. 3
C Architecture of the constructed Deep Neural Network 4
D Architecture of a stacked autoencoder 5
E ROC Curves for the different tested algorithms using the 10-fold test dataset 6
F ROC Curves for the different miRAW configurations using the 100-fold test dataset. 7
G Comparison of miRAW with different CSSMs and eight other common target prediction tools using the full test dataset. 8
H ROC Curves for the different tested algorithms using the full test dataset. 9

List of Tables

A Cross validation of miRAW's deep artificial neural network using XENT loss function. 10
B Cross validation of miRAW's deep artificial neural network using NLL loss function. 11
C Comparison of miRAW and other target prediction tools using the 10-fold test dataset 12
D Comparison of miRAW and other target prediction tools using the full test dataset 13

1 Supplementary Figures

1.1 Methods

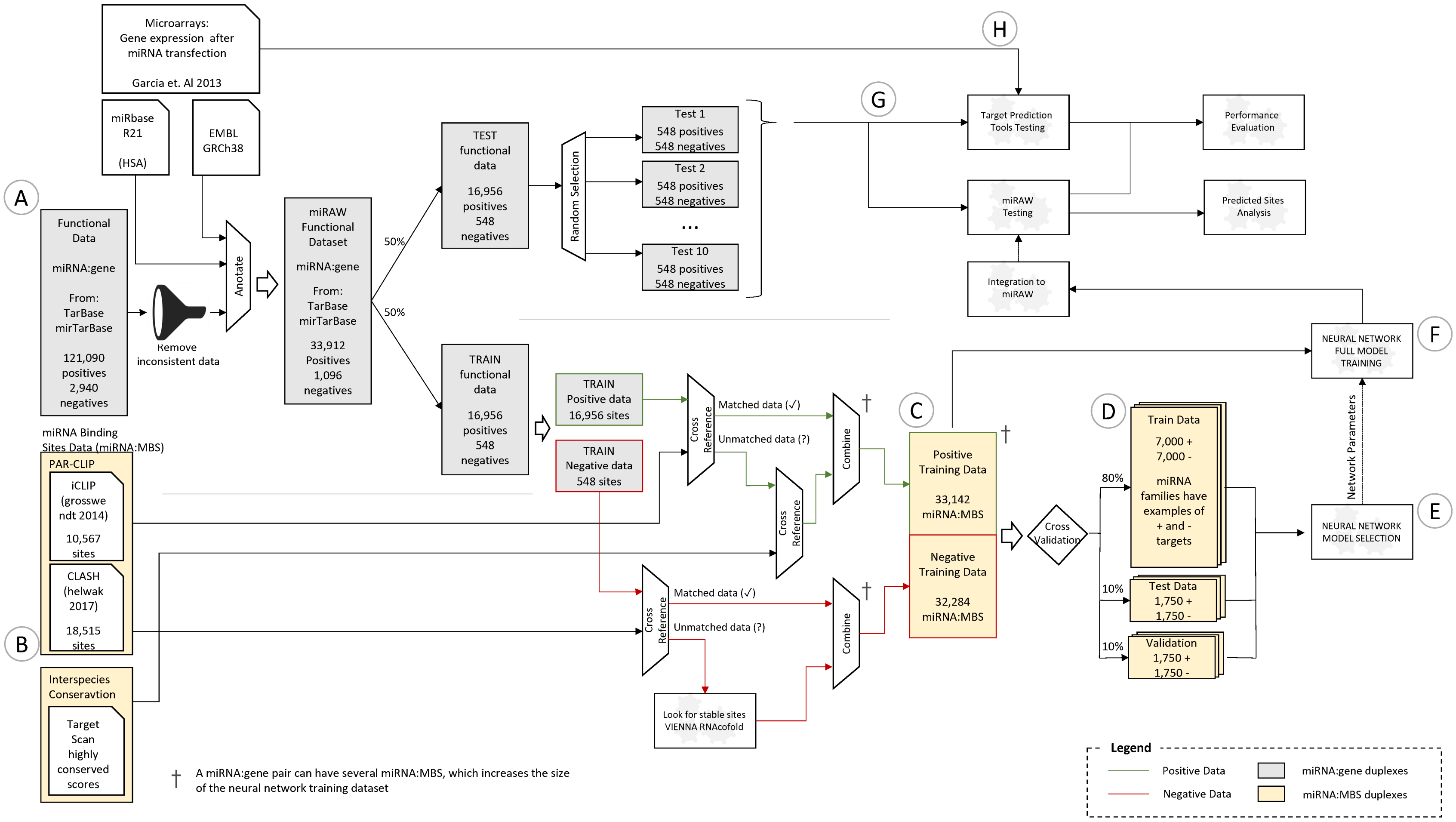

Fig A. Schematic of the procedure followed to generate training and testing data. Two types of datasets have been used to develop miRAW: (i) experimentally verified functional datasets that define if a miRNA targets a gene - in grey - and (ii) CLIP datasets that define miRNA binding site locations in the 3'UTR of a gene - in light yellow -. (A) TarBase and mirTarBase served as the principal functional datasets; after eliminating duplicated and/or inconsisten entries and annotating the miRNA:gene pairs using miRbase21 and the GRCh18 we divided the functional datasets into training and testing data. (B) Training data was cross referenced with CLIP data that provided potential miRNA binding sites for each miRNA:gene pair, thus for each miRNA:gene pair we obtained several miRNA:MBS duplexes; the positive training data was complemented using highly conserved MBS locations (obtained from target scan conservation scores) and the negative training data was complemented by selecting stable miRNA:MBS duplexes -provided by ViennaRNA- in experimentally validated negative miRNA:gene pairs. (C) The training data was split following a cross-validation approach (D) to select and tune the deep neural network; (F)after selecting the appropiate network design, we used the whole training dataset (balanced classes) to train miRAW's neural network. (G) The testing functional dataset was used to test miRAW and to compare it with other state-of-the-art target prediction tools. Additionally, we tested miRAW using independent experimental microarray datasets showing gene expression after transfection of specific miRNAs (Garcia et Al. 2011) to evaluate the relation between target prediction and actual miRNA repressive power.

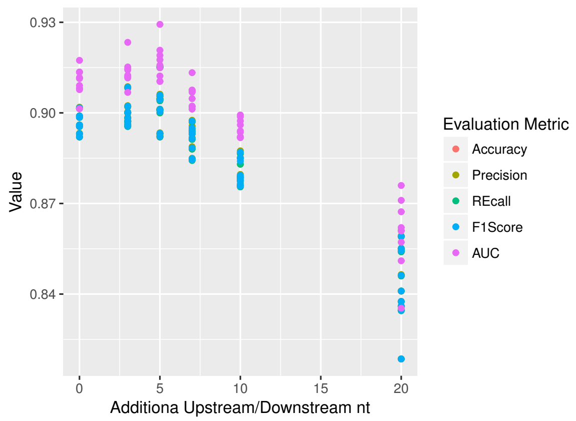

(a)

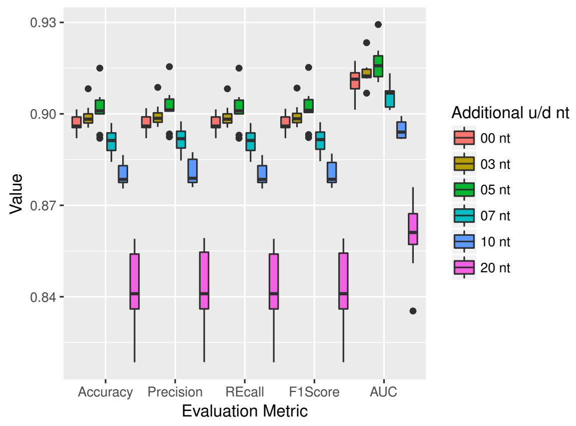

(b)

Fig B. The number of flanking upstream/downstream nucleotides conditions the performance of the network. To evaluate the number of flanking upstream/downstream nucleotides to be considered by the neural network we performed a 10-fold cross-validation analysis using a 10% of the training data and considering different number of nt -0, 3, 5, 7, 10 and 20-; being 5 the optimal parameter. (a)Scatter plot relating the number of considered upstream/downstream nucleotides and the perfomance of the neural network according to accuracy, precision, recall, f1-score and AUC metrics. The results show that considering 5 flanking nt results in the optimal configuration for miRAW. (b)Boxplot evaluating the number of considered upstream/downstream nucleotides and the perfomance of the neural network according to accuracy, precision, recall, f1-score and AUC metrics. The chart shows significant differences between the different configurations; showing that considering a some flanking nucleotides increases the network performance but that selecting too many can drop the network performance. Both charts point 5 as the optimal number of flanking nt to be considered as higher values result in significant lower performance.

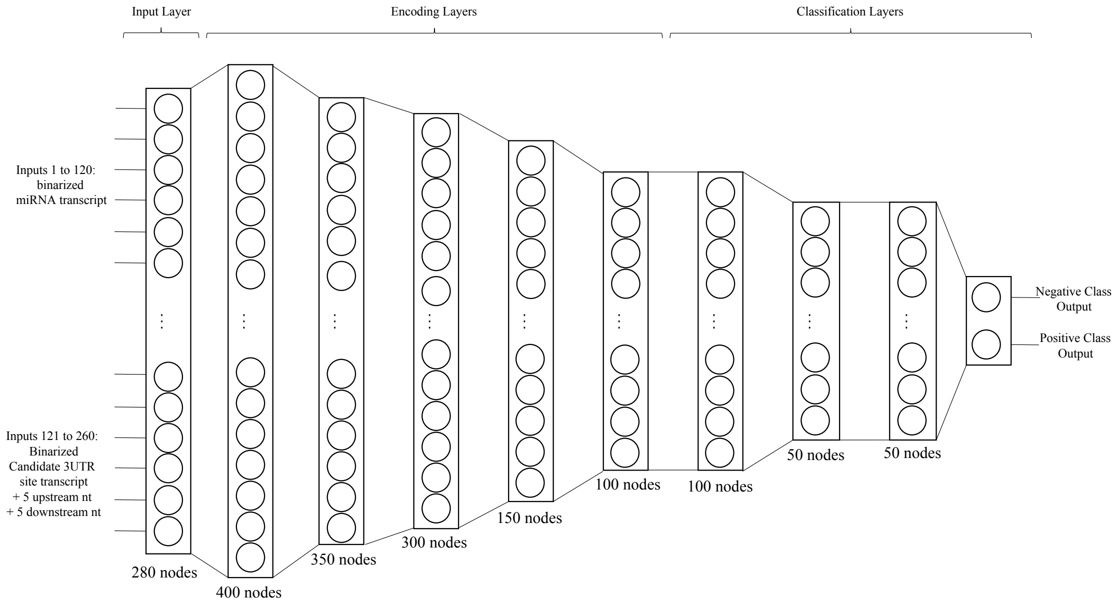

Fig C. Architecture of the constructed Deep Neural Network. The network is composed of eight dense hidden layers (comprising rectifier activation function -RelU-nodes) whilst the output layer comprises two softmax output nodes (one per possible class). The shape of the network is consistent with its intended functionality: (i) the first hidden sparse layer increases the dimensionality of the problem allowing the representation of data in a more complex dimension (over-completion). There is a debate in the machine learning community regarding the need of such over-completion layers, as they do not necessarily improve the efficiency of the network autoencoder whilst making the learning process slower. Considering that the relatively low number of inputs of the proposed network allows a fast training procedure, we opted to include an over-completion layer to give the network the chance of identifying more comlex pattern. (ii) Hidden layers one to five aim to identify the relevant features representing the data; they correspond to the first half of a stacked autoencoder. Those layers were pre-trained as an isolated autoencoder in order to learn the features that are most representative of miRNA:MBS duplexes. (iii) the last three layers are responsible for classifying the features learned by the autoencoder and follow the typical shape of a feedforward classification network.

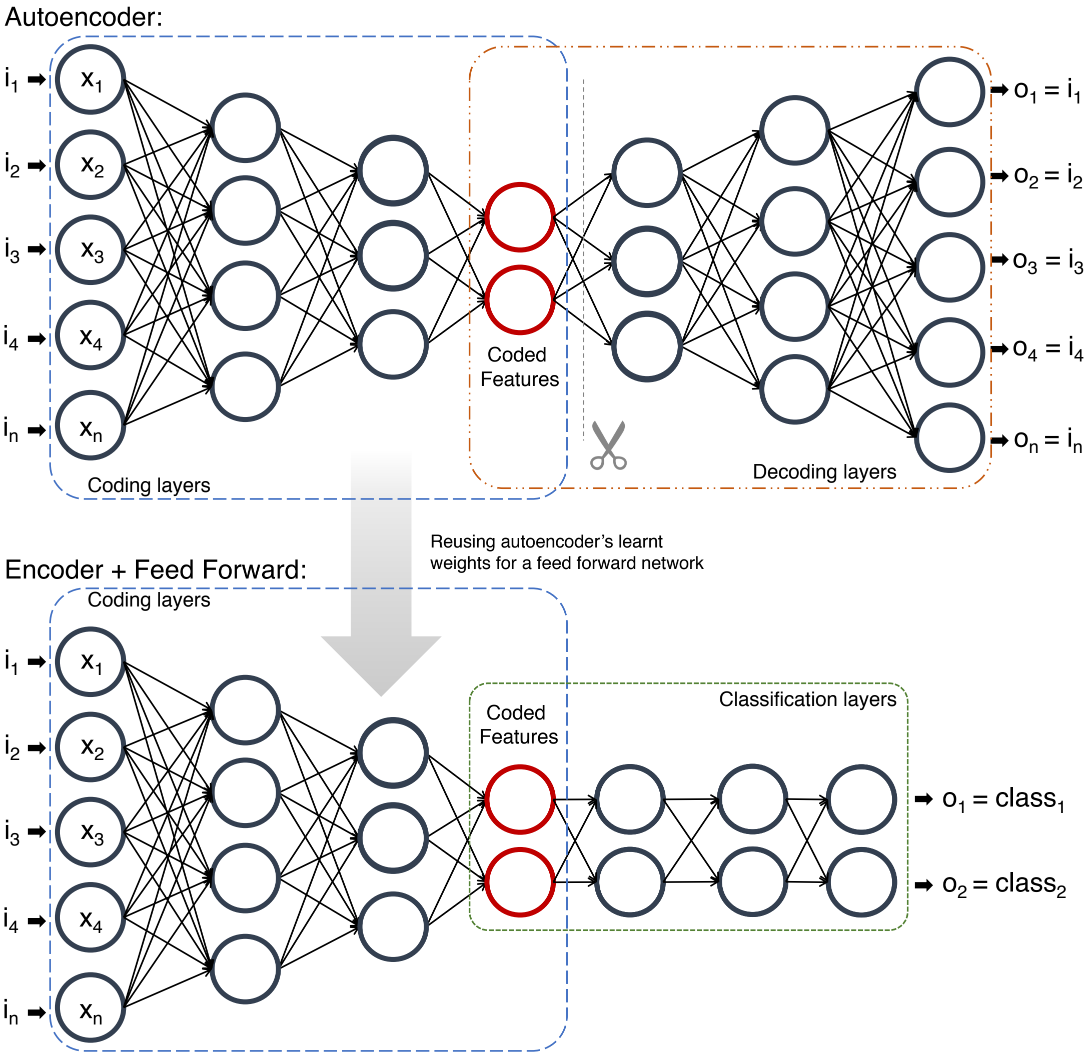

Fig D. Architecture of a stacked autoencoder. An autoencoder is an unsupervised artificial neural network used for dimensionality reduction and for automatically learning a set of features describing a complex data structure. An autoencoder has the same number of output as input nodes. Its purpose is to reconstruct the input values in the output nodes after compressing the input data into a lower dimensional space. An autoencoder (top figure) its composed of two parts: the coding layers – which compress the data – and the decoding layers – which reconstruct the compressed features –. In this work (i) we trained an autoencoder to learn the set of features representing miRNA:MBS interactions and (ii) combined the coding layers and their weights with a feed-forward neural network (bottom figure).

1.2 Results

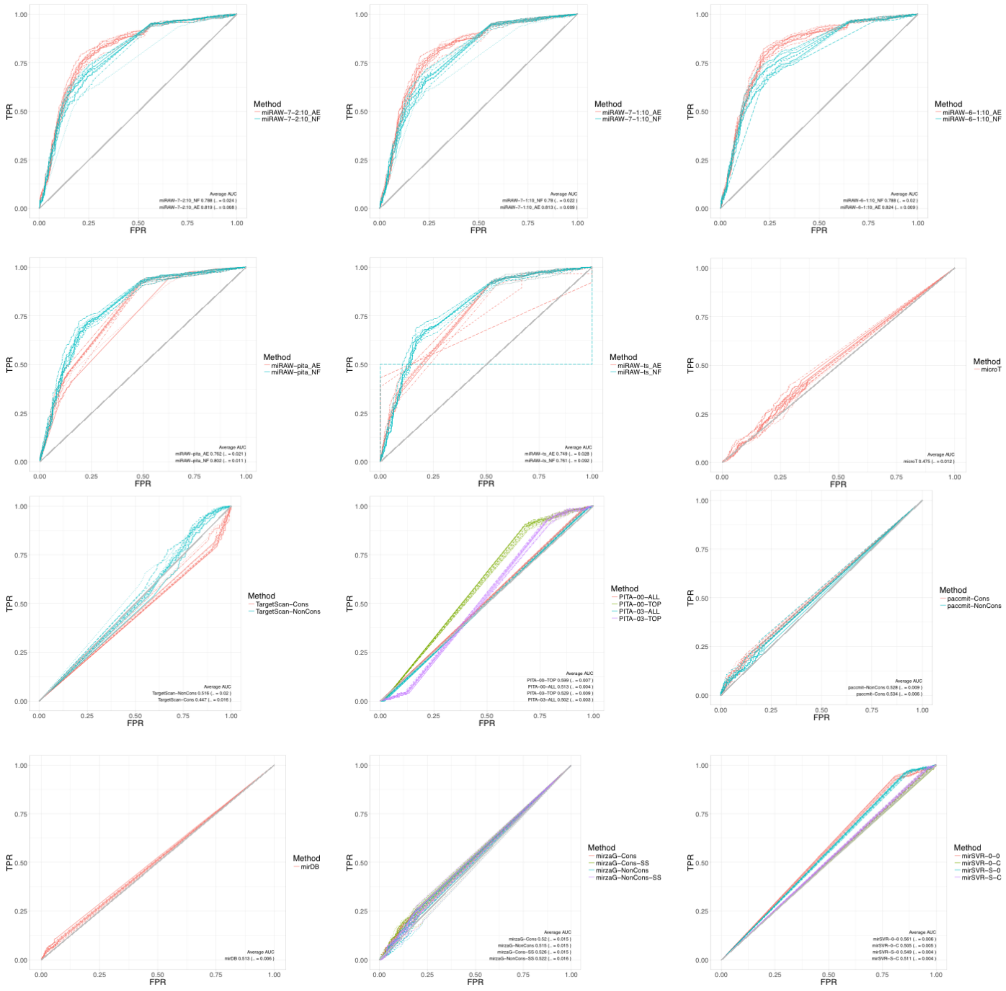

Fig E. ROC Curves for the different tested algorithms using the 10-fold test dataset. This plots show the ROC curves corresponding to the different algorithm predictions for each of the 10 folds of the test dataset. Different configurations of the same algorithm are plot using different colors. Generally miRAW methods obtain the best curves being miRAW-6-1:10 with Accessibility Energy filtering the method with the best results (AUC = 0.824, $\sigma = 0.009$). When using miRAW, CSSMs oriented to the prediction of non-canonical sites obtained better performances when combined with site accessibility energy filtering; on the other hand, more canonically oriented CSSMs (miRAW-ts and miRAW-pita) obtain better results when no a posteriori filtering is applied. Regarding the other tested methods PITA (release 00-top, AUC = 0.599) provides the best AUC, followed by mirSVR (AUC = 0.581). Note that most of the algorithm prediction datasets (microT, PITA, TargetScan and mirza-G) only include confident predictions with scores surpassing a certain established thresholds, this has a strong affect in the shape of the ROC curves and reduces the AUC values; making the AUCs not directly comparable with the ones from miRAW.

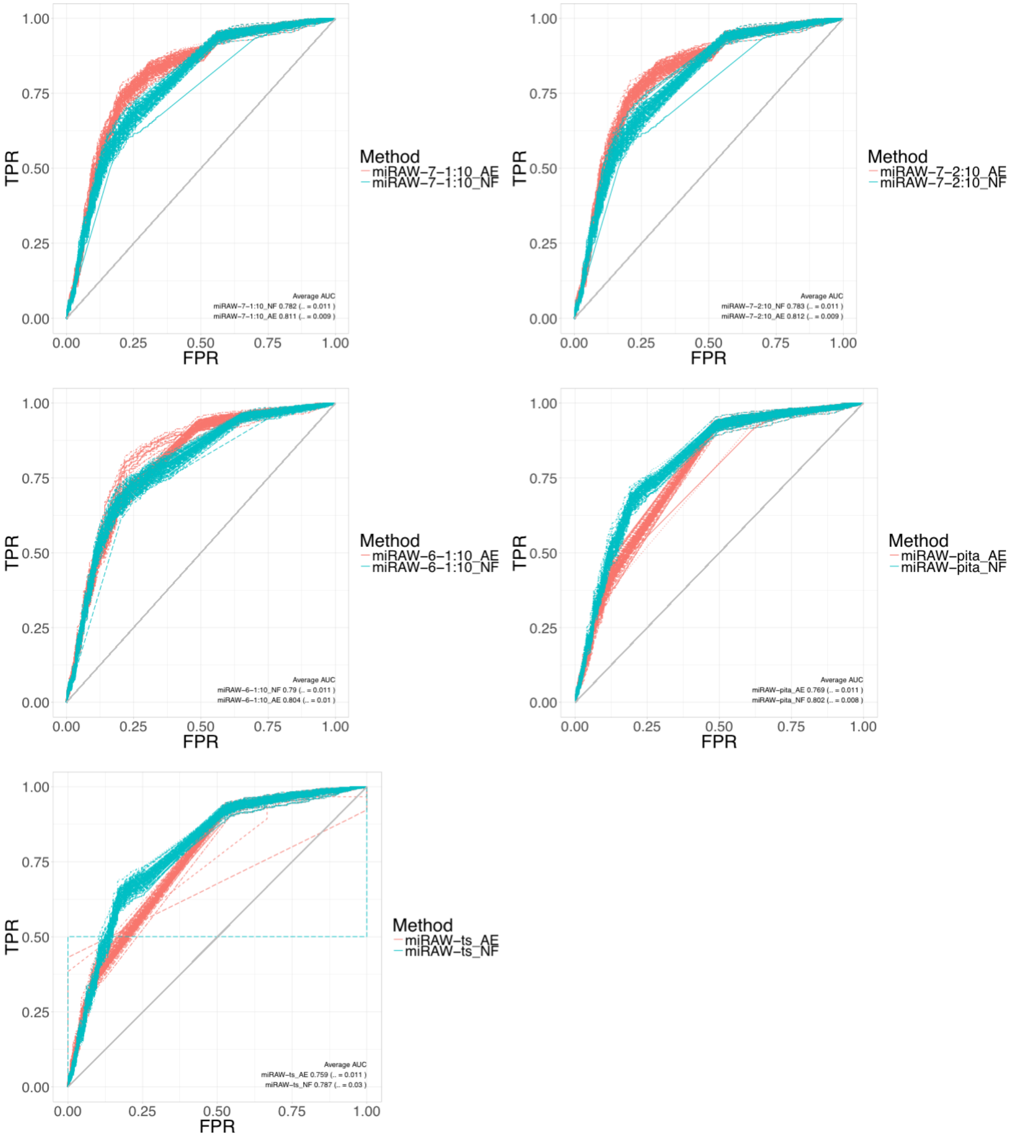

Fig F. ROC Curves for the different miRAW configurations using the 100-fold test dataset. The trend set when analyzing the 10-fold test dataset is also appreciated in this figure. The curves show how site accessibility energy filtering plays a different role for the different CSSMs. Non-canonical oriented methods (miRAW-7-1:10, miRAW-7-2:10 and miRAW-6-1:10) obtain better performance when applying post-filtering whilst more restrictive CSSMs ()miRAW-pita and miRAW-ts) perform better when no *a posteriori filtering* is applied.

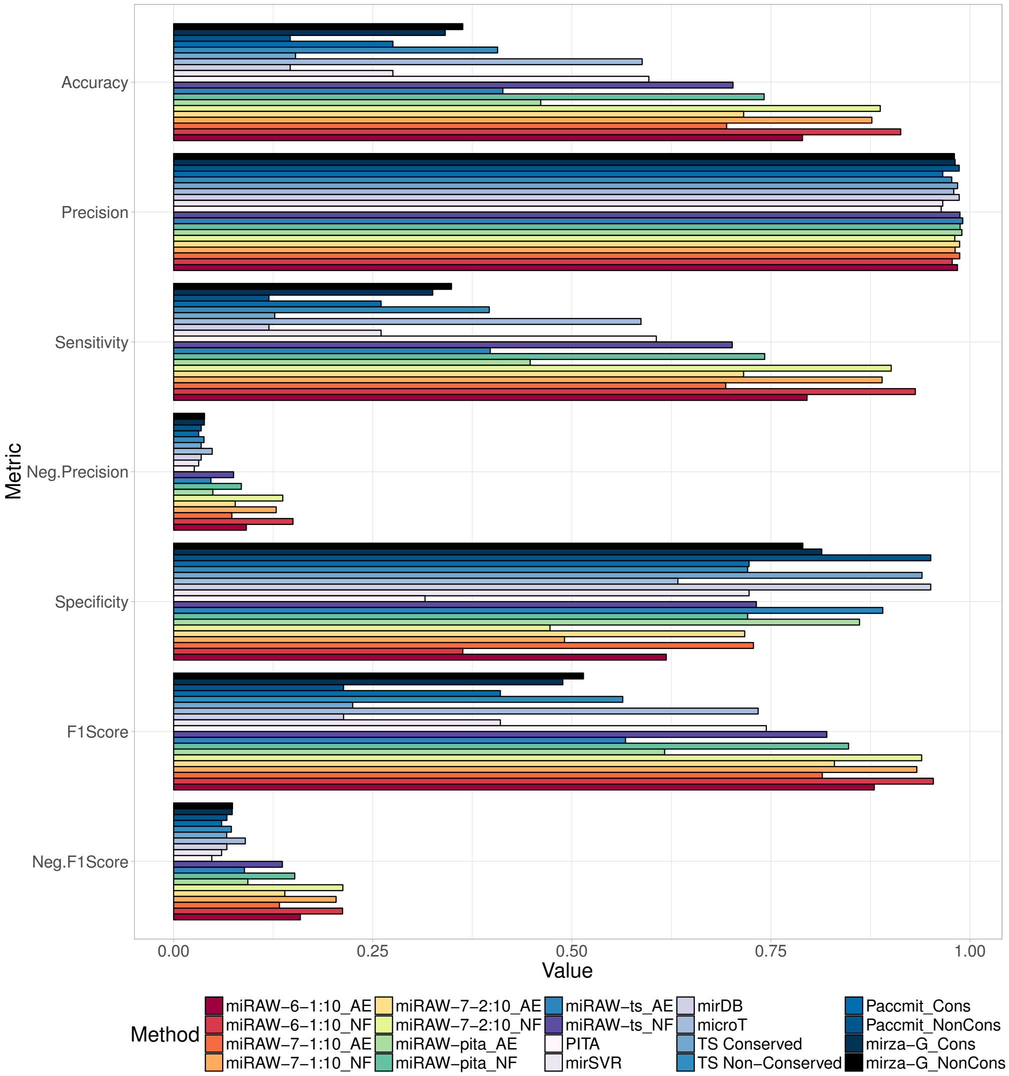

Fig G. Comparison of miRAW with different CSSMs and eight other common target prediction tools (TargetScan C & NC, Diana microT-CDS v4, PITA v6 , miRanda, Paccmit, mirzaG and mirDB) using the full test dataset. Colouring for miRAW results follow a rainbow pattern; other prediction tools follow a light to dark blue color schema. Evaluation was determined in terms of accuracy, precision, sensitivity, negative precision, specificity and F1-score (an ideal predictor would obtain a score of 1 for each metric). The optimal miRAW configurations outperformed other methods in terms of accuracy and F-scores, which are good representations of general performance measures; they also score amongst the top methods in the rest of metrics but negative precision. The unbalance in the test dataset (97:3 ratio between positive and negative data) favors methods biased towards the prediction of positive targets: algorithms following less restrictive approaches obtain higher specificity values whilst maintaining similar precision scores (PITA, microT-CDS); on the other hand, more conservative approaches (mirDB, mirSVR and TargetScan) obtain higher specificity and negative precision scores. After miRAW, microT was the method which presented better and more balanced results.

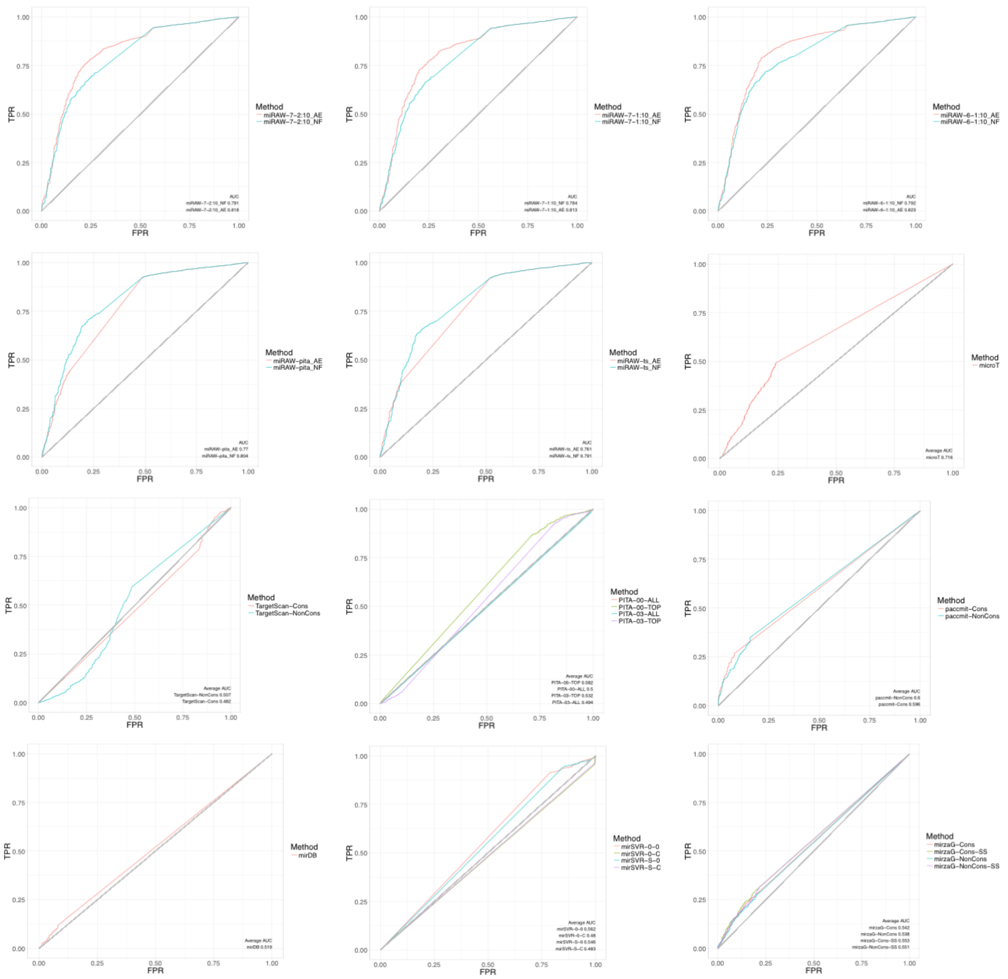

Fig H. ROC Curves for the different tested algorithms using the full test dataset. miRAW obtains the best results in terms of area under the curve (AUC) being miRAW-6-1:10AE (AUC = 0.823) the best configuration. The curves show how site accessibility energy filtering plays a different role for the different CSSMs: miRAW-7-1:10, miRAW-7-2:10 and miRAW-6-1:10 obtain better performance when applying post-filtering whilst miRAW-pita and miRAW-ts obtain worse results. Regarding the rest of the tested prediction methods, microT-CDS (AUC=0.716) and Pacmit-NonCons (0.6) emerge as the best non DL-based classifiers; their better performance is related to its balance regarding positive and negative predictions: whilst mirDB, targetScan, PITA and mirSVR follow restrictive approaches for site selection, microT-CDS and Paccmit have fewer constraints. Regarding the influence of interspecies conservation (Paccmit, TargetScan and Mirza-G), generally configurations that did not involved interspecies conservation information obtained slightly higher AUCs than configurations involving such information. Note that most of the algorithm prediction datasets (microT, PITA, TargetScan and mirza-G) only include confident predictions with scores surpassing a certain established thresholds, this has a strong affect in the shape of the ROC curves and reduces the AUC values; making the AUCs not directly comparable with the ones from miRAW. Suppleentary Table X shows the specific values for each score.

Supplementary Tables

Table A. Supplementary table 1 Cross validation of miRAW's deep artificial neural network results when using XENT loss function. The cross validation fold which obtained the best performance is highlighted in bold.

Fold	TN	FN	TP	FP	Accuracy	Precision	Sensitivity	Neg.Precision	Specificity	F1-Score	Neg. F1-Score	AUC
1	1698	291	1459	52	0.902	0.910	0.902	0.854	0.970	0.906	0.908	0.949
2	1555	105	1645	195	0.914	0.915	0.914	0.937	0.889	0.915	0.912	0.950
3	1671	221	1529	79	0.914	0.917	0.914	0.883	0.955	0.916	0.918	0.953
4	1559	71	1679	191	0.925	0.927	0.925	0.956	0.891	0.926	0.922	0.962
5	1622	141	1609	128	0.923	0.923	0.923	0.920	0.927	0.923	0.923	0.965
6	1696	226	1524	54	0.920	0.924	0.920	0.882	0.969	0.922	0.924	0.953
**7**	**1642**	**119**	**1631**	**108**	**0.935**	**0.935**	**0.935**	**0.932**	**0.938**	**0.935**	**0.935**	**0.968**
8	1643	136	1614	107	0.931	0.931	0.931	0.924	0.939	0.931	0.931	0.961
9	1643	157	1593	107	0.925	0.925	0.925	0.913	0.939	0.925	0.926	0.961
Average	1636.56	163.00	1587.00	113.44	0.921	0.923	0.921	0.911	0.935	0.922	0.922	0.958
Std. Dev.	51.82	69.58	69.58	51.82	0.010	0.008	0.010	0.032	0.030	0.009	0.009	0.007

Table B. Cross validation of miRAW's deep artificial neural network results when using NLL loss function. The cross validation fold which obtained the best performance is highlighted in bold.

Set	TN	FN	TP	FP	Accuracy	Precision	Sensitivity	Neg.Precision	Specificity	F1-Score	Neg. F1-Score	AUC
1	1586	142	1608	164	0.913	0.913	0.913	0.854	0.970	0.913	0.908	0.925
2	1566	143	1607	184	0.907	0.907	0.907	0.937	0.889	0.907	0.912	0.922
3	1603	142	1608	147	0.917	0.917	0.917	0.883	0.955	0.917	0.918	0.931
4	1587	118	1632	163	0.920	0.920	0.920	0.956	0.891	0.920	0.922	0.933
5	1555	139	1611	195	0.905	0.905	0.905	0.920	0.927	0.905	0.923	0.919
6	1581	139	1611	169	0.912	0.912	0.912	0.882	0.969	0.912	0.924	0.926
**7**	**1602**	**128**	**1622**	**148**	**0.921**	**0.921**	**0.921**	**0.932**	**0.938**	**0.921**	**0.935**	**0.934**
8	1577	137	1613	173	0.911	0.912	0.911	0.924	0.939	0.912	0.931	0.925
9	1577	133	1617	173	0.913	0.913	0.913	0.913	0.939	0.913	0.926	0.926
Average	1581.56	135.67	1614.33	168.44	0.913	0.913	0.913	0.911	0.935	0.913	0.922	0.927
Std. Dev.	15.48	8.19	8.19	15.48	0.006	0.005	0.006	0.032	0.030	0.006	0.009	0.005

Table C. Evaluation of the different miRAW configurations and different target prediction methods using the 10-fold testing dataset (TS stands for TargetScan).

method	TP	TN	FP	FN	Accuracy	Precision	Sensitivity	Neg.Precision	Specificity	F-Score	Neg.F-Score
miRAW-6-1:10_NF	**46.78 %**	18.16 %	31.84 %	03.22 %	0.649	0.595	**0.936**	**0.850**	0.363	0.727	0.509
miRAW-6-1:10_AE	39.64 %	30.94 %	19.06 %	10.36 %	0.706	0.675	0.793	0.750	0.619	0.729	0.678
miRAW-7-1:10_NF	44.51 %	24.45 %	25.55 %	05.49 %	0.690	0.635	0.890	0.817	0.489	0.741	0.612
miRAW-7-1:10_AE	34.69 %	36.41 %	13.59 %	15.31 %	0.711	0.718	0.694	0.704	0.728	0.706	0.716
miRAW-7-2:10_NF	45.26 %	23.54 %	26.46 %	04.74 %	0.688	0.631	0.905	0.833	0.471	**0.744**	0.601
miRAW-7-2:10_AE	36.09 %	35.86 %	14.14 %	13.91 %	0.719	0.718	0.722	0.721	0.717	0.720	0.719
miRAW-Pita_NF	36.89 %	36.04 %	13.96 %	13.11 %	**0.729**	0.725	0.738	0.733	0.721	0.731	**0.727**
miRAW-Pita_AE	22.52 %	43.07 %	06.93 %	27.48 %	0.656	0.764	0.450	0.611	0.861	0.566	0.715
miRAW-TS_NF	35.19 %	36.59 %	13.41 %	14.81 %	0.718	0.724	0.704	0.712	0.732	0.714	0.722
miRAW-TS_AE	19.76 %	44.53 %	05.47 %	30.24 %	0.643	**0.782**	0.395	0.596	0.891	0.525	0.714
TS Conserved	06.37 %	**46.99 %**	03.01 %	43.63 %	0.534	0.676	0.127	0.519	**0.940**	0.214	0.668
TS NonConserved	19.73 %	36.04 %	13.96 %	30.27 %	0.558	0.585	0.395	0.544	0.721	0.471	0.619
PITA	30.99 %	15.78 %	**34.22 %**	19.01 %	0.468	0.475	0.620	0.454	0.316	0.538	0.372
mirSVR	13.88 %	36.13 %	13.87 %	36.12 %	0.500	0.500	0.278	0.500	0.723	0.357	0.591
mirDB	06.20 %	47.54 %	02.46 %	**43.80 %**	0.537	0.714	0.124	0.520	0.951	0.211	0.673
microT	29.47 %	31.66 %	18.34 %	20.53 %	0.611	0.616	0.589	0.607	0.633	0.602	0.620
Paccmit_Cons	11.74 %	42.88 %	07.12 %	38.26 %	0.546	0.622	0.235	0.529	0.858	0.529	0.654
Paccmit_NonCons	08.39 %	45.80 %	04.20%	41.61 %	0.542	0.666	0.168	0.524	0.916	0.524	0.667
mirza-G_Cons	19.75 %	33.06 %	16.94 %	30.25 %	0.573	0.629	0.395	0.524	0.662	0.487	0.584
mirza-G_NonCons	18.26 %	35.00 %	15.00 %	31.74 %	0.559	0.620	0.365	0.524	0.700	0.465	0.600

Table D. Evaluation of the different miRAW configurations and different target prediction methods using the full testing dataset (TS stands for TargetScan).

	TP	FP	TN	FN	Accuracy	Precision	Sensitivity	Neg.Precision	Specificity	F-Score	Neg.F-score
miRAW-6-1:10_AE	76.98 %	1.23 %	1.99 %	19.80 %	0.790	0.984	0.795	0.091	0.619	0.880	0.159
miRAW-6-1:10_NF	90.14 %	2.05 %	1.17 %	6.64 %	0.913	0.978	0.931	0.150	0.363	0.954	0.212
miRAW-7-1:10_AE	67.10 %	0.87 %	2.34 %	29.68 %	0.694	0.987	0.693	0.073	0.728	0.815	0.133
miRAW-7-1:10_NF	86.11 %	1.64 %	1.58 %	10.68 %	0.877	0.981	0.890	0.129	0.491	0.933	0.204
miRAW-7-2:10_AE	69.27 %	0.91 %	2.31 %	27.51 %	0.716	0.987	0.716	0.077	0.717	0.830	0.140
miRAW-7-2:10_NF	87.21 %	1.70 %	1.52 %	9.57 %	0.887	0.981	0.901	0.137	0.473	0.939	0.212
miRAW-pita_AE	43.33 %	0.45 %	2.77 %	53.45 %	0.461	0.990	0.448	0.049	0.861	0.617	0.093
miRAW-pita_NF	71.84 %	0.90 %	2.32 %	24.95 %	0.742	0.988	0.742	0.085	0.721	0.848	0.152
miRAW-ts_AE	38.48 %	0.35 %	2.86 %	58.31 %	0.413	0.991	0.398	0.047	0.891	0.567	0.089
miRAW-ts_NF	67.89 %	0.86 %	2.35 %	28.89 %	0.702	0.987	0.701	0.075	0.732	0.820	0.137
TS Conserved	12.29 %	0.19 %	3.02 %	84.50 %	0.153	0.984	0.127	0.035	0.940	0.225	0.067
TS Non-Conserved	38.37 %	0.90 %	2.32 %	58.42 %	0.407	0.977	0.396	0.038	0.721	0.564	0.072
PITA	58.67 %	2.20 %	1.02 %	38.12 %	0.597	0.964	0.606	0.026	0.316	0.744	0.048
mirSVR	25.21 %	0.89 %	2.32 %	71.57 %	0.275	0.966	0.260	0.031	0.723	0.410	0.060
mirDB	11.58 %	0.16 %	3.06 %	85.20 %	0.146	0.987	0.120	0.035	0.951	0.213	0.067
microT	56.80 %	1.18 %	2.04 %	39.98 %	0.588	0.980	0.587	0.048	0.633	0.734	0.090
Paccmit_Cons	25.21 %	0.89 %	2.32 %	71.57 %	0.275	0.966	0.260	0.031	0.723	0.410	0.060
Paccmit_NonCons	11.58 %	0.16 %	3.06 %	85.20 %	0.146	0.987	0.120	0.035	0.951	0.213	0.067
mirza-G_Cons	31.49 %	0.60 %	2.62 %	65.29 %	0.341	0.981	0.325	0.039	0.814	0.489	0.074
mirza-G_NonCons	33.77 %	0.67 %	2.54 %	63.01 %	0.363	0.980	0.349	0.039	0.790	0.515	0.074
